# Medical Opinion and Movement

**Published:** 1910-10-08

**Authors:** 


### Medical Opinion and Movement.

### Diagnosis of Duodenal Ulcer.

In a contribution to the *Deutsche Medizinische Wochenschrift* Dr. Günzburg discourses on the signs and symptoms of duodenal ulcer. His experience is based upon eighteen cases, seventeen of which occurred in males, and in all he was able to confirm the diagnosis either by operation or by post-mortem examination. According to Moynihan and other British authorities the history of hunger pain coming on two or three hours after a meal and relieved by food is diagnostic of duodenal ulcer. These symptoms are generally rebellious to medical treatment, may persist for some weeks or months, and then disappear spontaneously to return again later. But Dr. Günzburg agrees with Ewald that this sort of history is not pathognomic of duodenal ulcer, and may occur in other morbid states, especially in conditions of arterial sclerosis. He lays emphasis on the occurrence of traces of blood in the fæces, and thinks that this sign, combined with the hunger pain, constitute a definite syndrome diagnostic of duodenal ulcer. A further sign of importance in a man between the ages of thirty and sixty is a diminution in the hæmoglobin content of the blood, and a fourth sign to which he draws attention and which appears hitherto to have escaped observation is the presence of a tympanitic area in the region of the quadrate lobe of the liver, due to a dilatation and distension of the duodenum with gas.

### Distension of the Pelvis during Confinement.

A somewhat novel method of obtaining a distension of the pelvis during confinement is described by Dr. King. The manœuvre was completely successful in the case reported by him, and it may prove of value in other similar cases, in which, owing to slight contraction of the pelvis or a rather large diameter of the fœtal head, difficulty is encountered in delivery. The patient, a multipara, had been in labour for six hours, and in spite of strong pains the head was fixed at the upper pelvic margin and failed to make any progress. The sagittal suture was transverse, and rather nearer the promontory than the pubis. The two fontanelles could be reached, and the anterior was a little lower than the posterior. The os had a diameter of 2 centimetres. The diagonal conjugate diameter measured 11 cm. and the fœtal biparietal 10 cm. The position of Walcher and the application of forceps failed to relieve the condition, and the author then had recourse to the following method: With the patient lying across the bed, the thighs separated, the three middle fingers of the right hand were introduced into the vagina and applied to the inner aspect of the right ischial tuberosity, so that the third finger was on the ischial insertion of the sacrosciatic ligament, the middle finger on the border of the obturator foramen and the index finger on the ascending ramus of the ischium. The left hand was then inserted and placed in a similar position to the left side. The forearms were thus crossed with the hands back to back. At each uterine contraction the hands were forced apart in the transverse diameter. After six pains (which appeared to the patient less painful than the preceding) in almost as many minutes, the head was completely engaged in the pelvis and the child was subsequently delivered without further assistance. The object of the method is, of course, to secure a little more room by a distension of the pelvic articulations.

### Senna as a Purgative.

Much good work has been done by the study of the effect of different purgatives upon the digestive tract by means of $x$-ray observations after a bismuth meal, and accounts of these observations have already appeared in these columns. Dr. Hertz, of Guy's Hospital, was one of the pioneers in this work, and his observations on the normal rate of progress of food through the stomach and intestinal tract and of the variations under different conditions are already well known. More recently Dr. E. Stierlin has investigated the effect of senna as a purgative by the same method of observation. An account of his work appears in the *Münchener Medizinische Wochenschrift*. His subjects for observation were three boys, aged eight, nine, and fifteen years respectively. The first thing in the morning 20 grammes of bismuth carbonate were given in 400 c.c. of warm water. The first meal was given two hours later and afterwards the usual meals during the rest of the day. Radiographic observations were made at intervals of two hours during the day. In the evening a soap-and-water enema was given and followed by a black stool. Two days later the same proceeding was repeated, but half-an-hour before the dose of bismuth carbonate 10 grammes of macerated senna pods were administered, and another 20 grammes at the same time as the bismuth dose. By comparing the two series of observation the author found that the senna only affects the peristaltic movements of the large intestine. It has little or no effect upon the movements of the stomach and small intestine. As soon as the senna reaches the cæcum and ascending colon it produces a complete evacuation of almost the whole contents of the colon, and this evacuation is repeated several times as long as any senna remains in the large intestine. The author therefore concludes from these observations that senna is an ideal purgative, causing, as it does, a complete evacuation of the lower bowel.

### Suture of the Recurrent Laryngeal Nerve.

A unique case of suture of the left recurrent laryngeal nerve is reported in the *Annals of Surgery* by Dr. J. Shelton Horsley. The patient was a negress aged forty, who had received a revolver shot in the neck. Gliding off the lower margin of the chin near the middle line, the shot had proceeded under the skin down the neck, had bruised the left side of the thyroid cartilage, and finally lodged in the root of the neck between the clavicle and external border of the trapezius muscle, where it was located by the $x$-rays. From the time of the accident the patient was unable to speak except in whispers, and respiration was difficult. She was first seen by Dr. Horsley two months after operation. Laryngoscopic examination showed complete paralysis of the left vocal cord. At the operation the shot was first extracted. An incision was made along the anterior margin of the sternomastoid muscle. The muscle and the carotid and jugular vessels were drawn outwards, and the left lobe of the thyroid, the larynx and trachea were drawn inwards. The recurrent laryngeal nerve was found divided just before its entrance into the larynx, where it was lost in a small mass of cicatricial tissue. The lacerated portion of 8 mm. length was excised and the ends sutured with fine catgut. The wound was closed and healed rapidly, but the immediate result was negative. But two months after operation a slight mobility of the left vocal cord was observed. Three months later its mobility was perfect. Phonation returned, but it lacked somewhat in fulness, which was due to slight adhesion between the anterior posterior of the two chords, due probably to long disuse and a certain degree of inflammation. Respiration became normal some time before the voice returned.

### The Microbic Cause of Rheumatism.

Since the original papers of Poynton and Paine some years ago most authorities have been content to allow to these observers the credit of having discovered the micrococcus which causes acute rheumatism, although there are still some pathologists who remain critical and unconvinced. In the *Lancet* the collaborators reaffirm their original contention that this coccus, which is sometimes a monococcus, sometimes a diplococcus, and sometimes a streptococcus, is the only existing and specific cause of acute rheumatism and of rheumatic endocarditis. They do not accept the view that it is merely a terminal infection, for some of those from whom it has been isolated are still living. It is certain that this organism can produce arthritis, carditis, and other rheumatic manifestations, although it is not found in every case examined. Further, their organism is capable of producing malignant as well as simple endocarditis, quite apart from any additional infection with pyogenic streptococci. As a matter of experience, in such cases it is often found that the micro-organism is in streptococcic form, but this is merely one of the characteristic phases in which it may appear, not a separate secondary infection. Investigations of the bacteriology of the tonsils has shown that in rheumatic patients with enlarged tonsils and recurring tonsilitis diplostreptococci predominate over the other flora of these organs. It is thence concluded that the tonsils not merely serve as an avenue of infection, but also as a means whereby relapses occur; and it is suggested that the tonsils should always be enucleated, if enlarged, in these subjects to prevent the source of infection being kept up.

### Physique and Intelligence.

The old Roman maxim about *mens sana in corpore sano* has already received some severe shocks at the hands of modern scientific research. The latest memoir (No. viii.) of the Eugenics Research Laboratory administers several more. In it Mr. David Heron exhaustively analyses results of school inspections and carefully allows for every disturbing factor whose influence he can trace. Into the higher mathematics of his elaborate tables it is unnecessary to follow him, but some of his conclusions are most interesting. Unless it is assumed, he says, that no reliance is to be placed on the teachers' classification into intelligence groups in the individual schools, there is really no basis for the belief that mental capacity is substantially associated with the height and weight of the children. It also emerges that the condition of the teeth, which might well be supposed to be some index of the general nutrition, is not associated in any marked degree with intelligence. As to clothing, it would be difficult, the report proceeds, to assert even that good clothing is a sign of good home environment, and that that is associated with intelligence. As for bodily nutrition, no relation can be established between this and mental capacity; but the roughness of the material and the difficulties of classification combine to make some of the co-efficients sensible as compared with their probable errors. Again, tonsils and adenoids do not seem to be associated with defective intelligence to any degree sufficient to make itself plainly evident statistically. The author concludes that there is little sensible effect of nurture, environment, and physique on intelligence. Some such effect probably exists, but is clearly so small that it cannot be the chief determining cause of the differentiation of intelligence.

### Association of Psoriasis with Jaundice.

The attention of many observers has been drawn to the way in which psoriasis is frequently associated with other diseases, generally systemic diseases of obscure origin. According to Hilbing, pulmonary tuberculosis, syphilis, diabetes, arthritis, and a neuropathic inheritance are among the commonest of these conditions. Gout, again, is a condition which some suppose to be unduly frequent among psoriatics, though Sir M. Morris disputes this. Gaucher ascribes it to the arthritic diathesis, a condition which he invokes to explain many other skin lesions whose etiology is still undetermined. On the other hand, many dermatologists believe it to have a parasitic origin; several parasites have been described, though none has as yet been acknowledged by the profession. In the *Bulletin of the Johns Hopkins Hospital* Dr. W. D. Higgins adds icterus to the list of toxæmias with which psoriasis has been observed to be associated. Two cases are reported. In one a woman of forty-five had a somewhat severe attack of apparently simple jaundice; when it subsided, a generalised diffuse psoriasis appeared, and lasted for three weeks. In the other a man of twenty-seven had simple jaundice for four weeks, and while still icteric was affected with psoriasis very extensively. Six weeks after the jaundice had subsided he was still suffering from psoriasis. Neither patient had ever had psoriasis or any other skin disease previously.

### Fibromyomata at Twelve Years Old.

The occurrence of uterine fibroids in juvenile patients is so rare that its occurrence has been questioned altogether by gynæcologists of experience. Mr. Bland Sutton has written: "There are few reliable records of fibroids being found in the uterus before the twentieth year." Winckel mentions but one case of fibroids before puberty, and other authorities have thrown doubt upon the diagnosis in the few reported cases. Dr. W. Hoare reports in the *Australasian Medical Gazette* the case of a child who began to lose blood per vaginam at eleven and a half years of age. The second period came on a fortnight after the first, and thence onwards she suffered from almost continual metrorrhagia. A month or two later an enlarged ovary was palpated, and proved on removal to be completely cystic and filled with sebaceous material. Hæmorrhage then ceased for four and a half months, after which it returned as bad as ever. A second laparotomy revealed that the other ovary, though small, was also cystic. The uterus was removed by supravaginal hysterectomy, and weighed 9 oz. The pathological report was that sections showed typical fibromyomatous structure. The patient made a rapid recovery and is now perfectly well. At the first onset of symptoms she had developed all the sexual characteristics of an adult woman. The case is, of course, most exceptional, but it shows that youth does not absolutely preclude a diagnosis of fibroids.

### Complete Treatment of Eclampsia.

The mortality rate of eclampsia in the United States is still about 33 per cent., according to Dr. B. C. Hirst. He believes that thorough treatment along the lines which he lays down in the *American Journal of Obstetrics and Gynæcology* will reduce this mortality below 10 per cent. Two ounces of castor oil with a minim or two of croton oil are introduced into the stomach with a tube; purgation is continued by 2-drachm doses of concentrated Epsom salts solution if the patient is able to swallow. Lavage of the stomach and colon are carried out. Diaphoresis is effected by thirty minutes in a sweat cabinet every four hours. After the first sweat a quart of salt solution is injected under the breasts, and subsequently at least as much per rectum after each sweat. If the blood-pressure is above 180 mm. of mercury, 16 oz. of blood are withdrawn by venesection. Fifteen minims of veratrum viride is given hypodermically; and afterwards $\frac{1}{100}$ grain of nitroglycerine every four hours. Chloral and chloroform are sometimes used for convulsions, but are regarded as unimportant. One grain of parathyroid extract is administered every four hours. If labour is going on, the membranes are punctured, but no form of *accouchement forcé* is allowed. Finally, treatment on this distinctly heroic scale is continued with lessening severity for a week after convulsions cease.

### Calcium Permanganate.

Dr. G. A. Stephens has long been prominent in his advocacy of various calcium salts for both external and internal therapeusis. In a paper published in the *Dublin Journal of Medical Science* he details his experience with a compound he has been recently trying, calcium permanganate. This substance occurs in purplish deliquescent crystals, readily soluble in water. The drug has, however, a most unpleasant taste and is therefore best ordered in capsules. The crystals, it must be borne in mind, readily attack the skin owing to their deliquescence, and must be handled carefully. For gastritis and gastric ulcer Dr. Stephens uses $\frac{1}{4}$-grain doses, and has had some remarkable successes in cases which seemed so desperate that even gastroenterostomy was held to be contraindicated; and in less severe cases he has been able to restore to complete health patients for whom this measure had been advised. For enteritis and colitis also it has, he thinks, considerable value. Empirically it has also been found useful for cases of malaria, and although the number of cases treated is small, yet the author is convinced that in this drug a valuable addition to the Pharmacopœia exists. As a local application it is recommended for rodent ulcer, inoperable offensive carcinoma, and similar conditions.

### Brudzinski's Signs.

Dr. Morse contributes to *Archives of Pediatrics* his findings with regard to the two reflexes described by Brudzinski in 1908 and 1909. The former of these is known as the contralateral reflex, and is a flexion of the leg elicited by passive flexion of the limb of the opposite side. The latter is known as the neck sign: passive flexion of the neck forwards causes flexion of the legs at the hips and knees, and a marked flexion of the legs on the pelvis. Brudzinski himself gave the frequency of the abnormal reflexes in cases of meningitis thus: neck sign, 97 per cent.; contralateral reflex, 66 per cent.; Kernig's sign, 57 per cent.; Babinski's sign, 50 per cent. Dr. Morse's conclusions confirm Brudzinski's researches. He says the contralateral reflex and the neck sign are never present in healthy infants and children, or in those ill with diseases other than those of the nervous system. They are almost never present in diseases of the nervous system other than meningitis. The contralateral reflex is found much less constantly than the neck sign. Both may occur intermittently or be absent throughout the whole course of the disease. Their presence in an acute disease is strong evidence in favour of meningitis; their absence does not exclude meningitis. The diagnostic value of the contralateral reflex is less than that of the neck sign. They occur in all types of meningitis and are of no importance in differentiating between them.

